# Supplementary material for: Content-rich biological network constructed by mining PubMed abstracts
Source: BMC Bioinformatics. 2004 Oct 8;5:147. doi: 10.1186/1471-2105-5-147 (PMC528731; doi:10.1186/1471-2105-5-147)
Supplement: Additional File 2 — The original results of the above study (non-essential files are deleted to keep the file size under the limit set by BMC bioinformatics). [file 1471-2105-5-147-S2.bz2 › chilibotAdditionalFile2/dip50/360ID9195914E5748/html/IL8_IL8RB.html]

 


 **IL8** and **IL8RB** 
  
Found 192 abstracts in PubMed, retrieved 50.  
 

 What does Google say? 
 PDF only 
| .edu only 

---

**Interactive relationship** (e.g. stimulation, inhibition, etc)

**Inhibitory relationship**- The selective CXCR2  [ **IL8RB** ]  antagonist SB272844 blocks interleukin 8  [ **IL8** ]  and growth related oncogene alpha mediated inhibition of spontaneous neutrophil apoptosis.  Ref: 12090783 Pulm Pharmacol Ther, 2002
- The pro survival effect of a fixed concentration of agonist IL 8  [ **IL8** ]  or Gro alpha on cultured neutrophils was abrogated by a selective CXCR2  [ **IL8RB** ]  antagonist SB272844 K D s 253 nM and 49.9 nM in the presence of IL 8  [ **IL8** ]  or Gro alpha respectively.  Ref: 12090783 Pulm Pharmacol Ther, 2002
- The induction of IL 8  [ **IL8** ]  in HAD, its inhibition of LTP, and the expression of its receptor, CXCR2  [ **IL8RB** ] , in the hippocampus all suggest that it plays a role in the cognitive dysfunction associated with HAD.  Ref: 12548717 J Neurosci Res, 2003
- After phagocytosis of opsonized yeast, the expression of CXCR1 and CXCR2  [ **IL8RB** ]  was substantially down regulated and was accompanied by reduced Ca responses to corresponding ligands, IL 8  [ **IL8** ]  and neutrophil activating peptide 2 NAP  [ **IL8** ]  2.  Ref: 12239185 Blood, 2002
**Neutral relationship**- IL 8  [ **IL8** ]  Induced Migratory Responses through CXCR1 and CXCR2  [ **IL8RB** ]  Association with Phosphorylation and Cellular Redistribution of Focal Adhesion Kinase.  Ref: 12627953 Biochemistry, 2003
- CXCR2  [ **IL8RB** ]  binds IL 8  [ **IL8** ]  and other structurally related chemokines such as growth related oncogene GRO a, GRObeta, GROgamma, neutrophil activating peptide 2 NAP  [ **IL8** ]  2 and epithelial cell derived neutrophil activating peptide 78 ENA 78.  Ref: 12365735 Fish Shellfish Immunol, 2002
- The migration activating concentrations of 50 ng mL GCP  [ **IL8** ]  2 and NAP  [ **IL8** ]  2 induced less potent migratory responses and FAK phosphorylation in CXCR2  [ **IL8RB** ]  expressing cells as compared with IL 8  [ **IL8** ] .  Ref: 12627953 Biochemistry, 2003
- Special emphasis is placed on the interaction of the human immunodeficiency virus HIV Tat protein with endothelial, astroglial, microglial, and neuronal cells, resulting in altered expression of vascular endothelial growth factor, fibroblast growth factor, interleukin 8  [ **IL8** ] , and regulation of calcium flux via CXCR2  [ **IL8RB** ] , which directly influences neuronal cell fitness.  Ref: 12476355 J Neurovirol, 2002
- Here, we show that survival of cultured astrocytes after Fas triggering is governed by the interaction of interleukin 8  [ **IL8** ]  with one of its receptors, CXCR2  [ **IL8RB** ] .  Ref: 12395092 Neuroreport, 2002
- Characterization of the molecular interactions of interleukin 8  [ **IL8** ]  CXCL8  [ **IL8** ] , growth related oncogen alpha CXCL1 and a non peptide antagonist SB 225002 with the human CXCR2  [ **IL8RB** ] .  Ref: 12628493 Biochem Pharmacol, 2003
- Angiogenic effects of interleukin 8  [ **IL8** ]  CXCL8  [ **IL8** ]  in human intestinal microvascular endothelial cells are mediated by CXCR2  [ **IL8RB** ] .  Ref: 12496258 J Biol Chem, 2003
- CXCR1 and CXCR2  [ **IL8RB** ]  mediate migratory activities in response to IL 8  [ **IL8** ]  and other ELR CXC chemokines e.g., GCP  [ **IL8** ]  2 and NAP  [ **IL8** ]  2.  Ref: 12627953 Biochemistry, 2003
- IL 8  [ **IL8** ]  or CXCL8  [ **IL8** ]  activates the receptors CXCR1 IL 8RA and CXCR2  [ **IL8RB** ]  IL 8RB to induce chemotaxis in leukocytes, but only CXCR1 mediates cytotoxic and cross regulatory signals.  Ref: 12626541 J Immunol, 2003
- IL 8  [ **IL8** ]  elicits angiogenic responses in microvascular endothelial cells isolated from human intestine by engaging CXCR2  [ **IL8RB** ] .  Ref: 12496258 J Biol Chem, 2003
- CXCL8  [ **IL8** ]  3 73 K11R G31P antagonizes ligand binding to the neutrophil CXCR1 and CXCR2  [ **IL8RB** ]  receptors and cellular responses to CXCL8  [ **IL8** ]  IL 8  [ **IL8** ] .  Ref: 12051749 Biochem Biophys Res Commun, 2002
- Furthermore, CXCL8  [ **IL8** ]  3 73 K11R G31P competitively antagonized the binding of CXCR1 and CXCR2  [ **IL8RB** ]  specific antibodies to these receptors.  Ref: 12051749 Biochem Biophys Res Commun, 2002
- CXCL8  [ **IL8** ]  exerts potent angiogenic properties on endothelial cells through interaction with its cognate receptors CXCR1 and CXCR2  [ **IL8RB** ] .  Ref: 12496258 J Biol Chem, 2003
- The effects of IL 8  [ **IL8** ]  and its related ligands are mediated via two receptors, CXCR1 and CXCR2  [ **IL8RB** ] .  Ref: 12444152 J Immunol, 2002
- The CXCR2  [ **IL8RB** ]  is a GPCR that is activated, amongst the others, by the chemokines CXCL8  [ **IL8** ]  IL 8  [ **IL8** ]  and CXCL2 GROb to induce cell chemotaxis.  Ref: 12888558 J Biol ChemJ Biol Chem, 2002
- We recently engineered a series of high affinity CXCL8  [ **IL8** ]  IL 8  [ **IL8** ]  antagonists, one of which, CXCL8  [ **IL8** ]  3 73 K11R G31P, binds very strongly to neutrophils via the CXCR1 and CXCR2  [ **IL8RB** ] .  Ref: 12406656 Vet Immunol Immunopathol, 2002
- As CXCR1 and CXCR2  [ **IL8RB** ]  expression is differentially regulated in tissue specific endothelial cells and effects of IL 8  [ **IL8** ]  on intestinal endothelial cells are not defined, we characterized the potential IL 8  [ **IL8** ]  induced angiogenic mechanisms in primary cultures of human intestinal microvascular endothelial cells HIMEC and IL 8  [ **IL8** ]  receptor expression in human intestinal microvessels.  Ref: 12496258 J Biol Chem, 2003

**Non-interactive relationship** (e.g. studied together, co-existance, homology, etc.)

- Thus in COPD, in addition to CXCL8  [ **IL8** ]  and CXCR1, CXCL5 and CXCR2  [ **IL8RB** ]  appear to play important roles in the airway neutrophilia characteristic of severe exacerbations.  Ref: 12857718 Am J Respir Crit Care MedAm J Respir Crit Care Med, 2003
- Standard immunohistochemical and immunocytochemical techniques were utilized in order to localize IL 8  [ **IL8** ]  and its receptors CXCR 1 and CXCR 2  [ **IL8RB** ]  in archival gingival specimens eight periodontitis and four non inflamed controls and in cultured gingival keratinocytes.  Ref: 12009185 J Periodontal Res, 2002
- Neutrophils throughout the synovium bound CXCL8  [ **IL8** ]  but did not show any signal for binding CCL2, CCL3 or CCL5.Immunohistochemistry showed that both CXCR1 and CXCR2  [ **IL8RB** ]  are expressed by macrophages and neutrophils in the rheumatoid and nonrheumatoid synovia, suggesting that both of these receptors are responsible for the CXCL8  [ **IL8** ]  binding.  Ref: 12010572 Arthritis Res, 2002
- However, the contribution of polymorphisms of the IL 8  [ **IL8** ]  gene and its receptor genes CXCR 1 and CXCR 2  [ **IL8RB** ]  to human TB susceptibility remains untested.  Ref: 12870115 J Infect Dis, 2003
- In this study, we addressed the question of whether expression of the interleukin 8  [ **IL8** ]  receptors IL 8RA CXCR1 and IL 8RB CXCR2  [ **IL8RB** ]  is upregulated in H. pylori infected human gastric biopsy samples.  Ref: 12761119 Infect Immun, 2003
- IL 8  [ **IL8** ]  activation of CXCR1, but not the IL 8  [ **IL8** ]  receptor CXCR2  [ **IL8RB** ] , cross phosphorylated CCR5 and CXCR4 and cross desensitized their responsiveness to RANTES regulated on activation normal T cell expressed and secreted CCL5 and stromal derived factor SDF 1 or CXCL12, respectively.  Ref: 12594210 J Biol Chem, 2003
- The immunohistochemical analysis using specific antibodies demonstrated that positive staining for IL 8  [ **IL8** ] , CXCR1 and CXCR2  [ **IL8RB** ]  in surgically resected human pancreatic cancer was 5 5 and 65%, respectively.  Ref: 12632066 Int J Oncol, 2003
- Cloning of the IL 8  [ **IL8** ]  receptor is important to study the function of IL 8  [ **IL8** ]  CXCR1 and CXCR2  [ **IL8RB** ]  in inflammation and signal transduction in fish.  Ref: 12365735 Fish Shellfish Immunol, 2002
- Thus far IL 8  [ **IL8** ]  and CXCR1 and CXCR2  [ **IL8RB** ]  proteins from rainbow trout have not been reported however, the sequence of a rainbow trout IL 8  [ **IL8** ]  has been noted GenBank Accession No.  Ref: 12365735 Fish Shellfish Immunol, 2002
- By comparison with the NSC and S COPD groups, neutrophilia and gene expression for epithelial derived neutrophil attractant 78 CXCL5, interleukin 8  [ **IL8** ]  CXCL8  [ **IL8** ] , CXCR1 and CXCR2  [ **IL8RB** ]  were each up regulated in the E COPD group p&lt.  Ref: 12857718 Am J Respir Crit Care MedAm J Respir Crit Care Med, 2002
- Furthermore, interleukin 8  [ **IL8** ]  secretion and CXCR2  [ **IL8RB** ]  expression are both induced in human astrocytes after Fas stimulation, suggesting a new mechanism of self defence against Fas mediated death.  Ref: 12395092 Neuroreport, 2002
- OBJECTIVE We sought to investigate the in vivo effect of bradykinin on the expression of the IL 8  [ **IL8** ]  receptors CXCR1 and CXCR2  [ **IL8RB** ]  in nasal cells.  Ref: 12532104 J Allergy Clin Immunol, 2003
- ... expression of the IL 8  [ **IL8** ]  receptor CXCR2  [ **IL8RB** ]  was reciprocally up regulated as detected by RT PCR.  Ref: 12244149 J Immunol, 2002
- We examined the expression of IL 8  [ **IL8** ]  and its two receptors CXCR1 and CXCR2  [ **IL8RB** ]  in 40 surgically resected human pancreatic cancer tissues and in three different human pancreatic cancer cell lines PANC 1, MIAPaCa 2 and Capan 2.  Ref: 12632066 Int J Oncol, 2003
- Expression of the interleukin 8  [ **IL8** ]  receptors CXCR1 and CXCR2  [ **IL8RB** ]  on cord blood derived cultured human mast cells.  Ref: 12065915 Int Arch Allergy Immunol, 2002
- Two IL 8  [ **IL8** ]  receptors, CXCR1 and CXCR2  [ **IL8RB** ] , have been identified in mammals 3 6.  Ref: 12365735 Fish Shellfish Immunol, 2002
- The stimulation of CXCR1 and CXCR2  [ **IL8RB** ]  by IL 8  [ **IL8** ]  concentrations that result in migratory activation induced focal adhesion kinase FAK phosphorylation in a G alpha i dependent manner.  Ref: 12627953 Biochemistry, 2003
- Preincubation of hippocampal slices with a monoclonal CXCR2  [ **IL8RB** ]  antibody 2 microg ml but not control IgG 2 microg ml blocked IL 8  [ **IL8** ]  induced inhibition of LTP.  Ref: 12548717 J Neurosci Res, 2003
- Because the chemotactic responses of neutrophils are impaired after phagocytosis, we explored the effect of phagocytic stimuli on the expression of interleukin 8  [ **IL8** ]  IL 8  [ **IL8** ]  receptors, CXCR1 and CXCR2  [ **IL8RB** ] , in human neutrophils.  Ref: 12239185 Blood, 2002
- The presence of putative factors that delay neutrophil apoptosis was investigated using inhibitors to leukotriene B4, lipopolysaccharide and the IL 8  [ **IL8** ]  receptor CXCR2  [ **IL8RB** ] , and blocking antibodies to granulocyte monocyte colony stimulating factor.  Ref: 12358343 Eur Respir J, 2002
- The mutants generated showed that a for CXCL8  [ **IL8** ]  binding, the N terminus of CXCR1 and the second extra cellular loop of CXCR2  [ **IL8RB** ]  are determinant, the N terminus of CXCR2  [ **IL8RB** ]  is not sufficient and the transmembrane domain seven is probably involved.  Ref: 12628493 Biochem Pharmacol, 2003
- In the present study, we examined the expression of chemokine receptors CXCR1 and CXCR2  [ **IL8RB** ]  receptors for interleukin 8  [ **IL8** ]  and CCR5 receptor for RANTES regulated on activation, normal T cell expressed and secreted, macrophage inflammatory protein MIP 1alpha, and MIP 1beta in human endometrium.  Ref: 12606476 Biol Reprod, 2003
- There are at least two different IL 8  [ **IL8** ]  receptor types CXCR1 and CXCR2  [ **IL8RB** ] .  Ref: 12561382 Sheng Wu Yi Xue Gong Cheng Xue Za Zhi, 2002
- RESULTS Expression of IL 8  [ **IL8** ]  receptors CXCR1 and CXCR2  [ **IL8RB** ]  was demonstrated by flow cytometry and of both mRNA by RT PCR.  Ref: 12065915 Int Arch Allergy Immunol, 2002
- In vitro, the antagonist potently inhibited human IL 8  [ **IL8** ]  induced chemotaxis of rabbit neutrophils IC 50 = 0.75 nM, suggesting that inhibition of leukocyte migration into the knee joint is a likely mechanism by which the CXCR2  [ **IL8RB** ]  antagonist modulates disease.  Ref: 12444152 J Immunol, 2002
- Expression of the chemokine receptors CCR2, specific for MCP 1, and CXCR1 and CXCR2  [ **IL8RB** ] , specific for IL 8  [ **IL8** ] , were not altered by HCMV infection.  Ref: 12714640 Invest Ophthalmol Vis Sci, 2003
- Human embryonic kidney HEK 293 cells expressing recombinant G alpha i coupled, human CXC chemokine receptor 2 CXCR2  [ **IL8RB** ]  were used to study the elevation of the intracellular Ca 2 Ca 2 i in response to interleukin 8  [ **IL8** ]  IL 8  [ **IL8** ]  following pre stimulation of endogenously expressed P2Y1 or P2Y2 nucleotide receptors.  Ref: 11877327 Br J Pharmacol, 2002
- Transendothelial migration of neutrophils in response to CXCL7 or IL 8  [ **IL8** ]  CXCL8  [ **IL8** ]  was subject to modulation by CTAP III, but not CXCL4, as seen by drastic desensitization of the migratory response of neutrophils pre exposed to CTAP III, which was paralleled by selective down modulation of CXCR 2  [ **IL8RB** ] .  Ref: 12193731 J Immunol, 2002
- Neutralizing antibodies to CXCR2  [ **IL8RB** ]  diminished IL 8  [ **IL8** ]  induced chemotaxis and stress fiber assembly.  Ref: 12496258 J Biol Chem, 2003
- A self defence mechanism of astrocytes against Fas mediated death involving interleukin 8  [ **IL8** ]  and CXCR2  [ **IL8RB** ] .  Ref: 12395092 Neuroreport, 2002
- Our data suggest that IL 8  [ **IL8** ]  directly enhanced endothelial cell proliferation, survival, and MMP expression in CXCR1 and CXCR2  [ **IL8RB** ]  expressing endothelial cells and regulated angiogenesis.  Ref: 12626597 J Immunol, 2003
- The aims of this study were to investigate the effects of Interleukin 8  [ **IL8** ]  IL 8  [ **IL8** ]  and Growth related oncogene alpha Gro alpha on neutrophil apoptosis and determine the potential for a selective CXCR2  [ **IL8RB** ]  antagonist to inhibit these responses.  Ref: 12090783 Pulm Pharmacol Ther, 2002
- Consistent with this conclusion is inhibition of the chemotactic response to monocytes by agents that block CXCR2  [ **IL8RB** ] , the receptor that NAP  [ **IL8** ]  2 uses.  Ref: 12101278 J Leukoc Biol, 2002
- Both IL 8  [ **IL8** ]  receptors, CXCR1 and CXCR2  [ **IL8RB** ] , are expressed extensively in the fetal intestine.  Ref: 12633567 Cytokine, 2002
- In the present study, we demonstrate that a potent and selective nonpeptide antagonist of human CXCR2  [ **IL8RB** ]  potently inhibits 125 I labeled human IL 8  [ **IL8** ]  binding to, and human IL 8  [ **IL8** ]  induced calcium mobilization mediated by, rabbit CXCR2  [ **IL8RB** ]  IC 50 = 40.5 and 7.7 nM, respectively, but not rabbit CXCR1 IC 50 = >1000 and 2200 nM, respectively.  Ref: 12444152 J Immunol, 2002
- Analysis of inter relationships between CXCR1 and CXCR2  [ **IL8RB** ]  during internalization indicated that the exposure of cells that expressed both CXCR1 and CXCR2  [ **IL8RB** ]  to CXCL8  [ **IL8** ]  or CXCL6 resulted in decreased levels of CXCR1 internalization as compared to those in cells that expressed only CXCR1.  Ref: 12442335 Eur J Immunol, 2002
- In contrast, in CXCR2  [ **IL8RB** ]  RBL cells the migration attenuating concentrations of IL 8  [ **IL8** ]  induced promoted levels of FAK phosphorylation and different patterns of FAK phosphorylation on its six potential tyrosine phosphorylation sites, as compared to activating concentrations of the chemokine.  Ref: 12627953 Biochemistry, 2003
- Biologic features, including IL 8  [ **IL8** ]  production, DNA binding activity, transactivation of NF kappaB, cell growth activity, cell viability, and the expression of IL 8  [ **IL8** ]  receptors CXCR1 and CXCR2  [ **IL8RB** ]  were analyzed.  Ref: 12216086 Cancer, 2002
- Angiostatin blocked chemotaxis of neutrophils to CXCR2  [ **IL8RB** ]  chemokine receptor agonists IL 8  [ **IL8** ] , MIP 2, and GROalpha, formyl Met Leu Phe fMLP, and 12 O tetradecanoylphorbol 13 acetate, and repressed fMLP induced mitochondrial activity.  Ref: 11772950 FASEB J, 2002
- Finally, the mechanism of binding of a non peptide antagonist on CXCR2  [ **IL8RB** ]  was investigated it occurred through epitopes a which were disseminated within the receptor, b which differed according to the use of CXCL8  [ **IL8** ]  or CXCL1 as a competitor and c which did not necessarily overlap with agonist binding sites.  Ref: 12628493 Biochem Pharmacol, 2003
- Our results support the hypothesis that opioids play a significant role in the susceptibility of the CNS to HIV 1 infection and subsequent encephalopathy by inhibiting local production of HIV 1 protective chemokines IL 8  [ **IL8** ]  and macrophage inflammatory protein 1beta and enhancing expression of HIV 1 entry coreceptor genes CCR3, CCR5, and CXCR2  [ **IL8RB** ]  within the CNS.  Ref: 12244149 J Immunol, 2002
- The presence of wortmannin during receptor recycling inhibited CXCR1 and CXCR2  [ **IL8RB** ]  re expression following CXCL8  [ **IL8** ]  induced internalization, and resulted in a marked disruption of the proper organization of actin filaments.  Ref: 12442335 Eur J Immunol, 2002
- Supported by the notion that malignant colonic epithelial cells overexpress IL 8  [ **IL8** ] , CXCR2  [ **IL8RB** ]  blockade may be a novel target for anti angiogenic therapy in colorectal adenocarcinoma.  Ref: 12496258 J Biol Chem, 2003
- It is well known that chemokines and chemokine receptors, such as interleukin 8  [ **IL8** ]  and its receptors CXCR1 and CXCR2  [ **IL8RB** ] , play important roles in the pathogenesis of psoriasis.  Ref: 12823305 Clin Exp Dermatol, 2003
- Human CMV UL146 encodes a neutrophil attracting chemokine denoted viral CXC chemokine 1 vCXCL1 that is as potent as host IL 8  [ **IL8** ]  and functions via the CXCR2  [ **IL8RB** ]  receptor, one of two human IL 8  [ **IL8** ]  receptors.  Ref: 12224512 Curr Top Microbiol Immunol, 2002
- The investigation of expression in IL 8  [ **IL8** ]  receptors, CXCR1 and CXCR2  [ **IL8RB** ] , revealed that the expression of both receptors was enhanced remarkably by curcumin.  Ref: 12216086 Cancer, 2002
